# Positive mental health and sense of coherence among emergency medical service professionals

**DOI:** 10.3389/fpubh.2024.1344872

**Published:** 2024-02-20

**Authors:** Susana Mantas-Jiménez, Glòria Reig-García, Marta Roqueta-Vall-Llosera, David Camara-Liebana, Afra Masià-Plana, Maria Teresa Lluch-Canut, Dolors Juvinya-Canal

**Affiliations:** ^1^Department of Nursing, University of Girona, Girona, Spain; ^2^Research Group Health and Healthcare, University of Girona, Girona, Spain; ^3^Health Gender and Aging Research Group, Girona, Spain; ^4^Department of Psychology, Quality of Life Research Institute, Universitat de Girona, Girona, Spain; ^5^Mental Health Sciences Department, School of Nursing, University of Barcelona, Barcelona, Spain

**Keywords:** positive mental health, sense of coherence, salutogenic model, emergency medical services, healthcare workers

## Abstract

**Background:**

Positive mental health (PMH) is a construct used to define and evaluate health from a positive perspective. Healthcare professionals in the emergency ambulance service are more likely to experience mental health disorders than the overall population. The demographic and occupational variables and Sense of Coherence (SOC) can act as predictors of PMH and can serve as protective elements against stress and demanding situations in the work environment.

**Objective:**

This study aimed to evaluate PMH and its relationship with demographic and occupational variables and determine if SOC is a predictive variable for PMH in health professionals working in the emergency ambulance service.

**Methods:**

A descriptive, cross-sectional, and correlational study was conducted with a sample of 406 healthcare professionals from the emergency ambulance service in eight health regions of Catalonia, Spain. The following variables were analyzed: Age, biological sex, household members, dependents family members, professional category, type of contract, job satisfaction and sense of collaboration with other institutions. The following assessment instruments were used: Positive Mental Health Questionnaire and Sense of Coherence scale.

**Results:**

High scores were obtained in Positive Mental Health (PMH). Study participants who reported feeling completely satisfied in their work also showed a significant relationship with all PMH factors: greater satisfaction with their personal life, in their helpful prosocial attitude toward others, in their capability to deal effectively with stress and navigate conflict scenarios, in their ability to solve problems and self-realization, greater empathy and ability to understand the feelings of others, greater ability to establish interpersonal relationships. Comprehensibility, as a dimension of SOC, was identified as a predictor for some factors of PMH: a greater personal satisfaction, self-control, autonomy, interpersonal skills and total PMHQ. More than 43% of positive mental health in health professionals is explained by higher Meaningfulness and Comprehensibility values of the SOC, the absence of dependent family members and having a non-graduate background.

**Conclusion:**

Healthcare workers in the emergency ambulance service had high PMH. Meaningfulness (ME) and Comprehensibility (C), dimensions of SOC, were identified as model predictors of greater PMH, showing higher scores in most of PMH factors. To enhance SOC as a mental health promotion measure, resilience programs should be implemented to help professionals develop skills to face and overcome adverse situations. Educating in stress management thought networks are key elements to strengthen SOC. Managers in emergency medical services play a key role in transforming healthcare work environments to promote positive outcomes in the mental health of their healthcare workers.

## Introduction

The promotion of mental health is one of the main health challenges of the last decades. Pandemics, wars and natural disasters has underscored the understanding that mental health is equally crucial as physical well-being, with both frequently exhibiting empirical connections. As a result, health plan administrators worldwide should considerer this information (1). Promoting Positive Mental Health (PMH) is an inherent part of public health. The World Health Organization (WHO) placed special emphasis on the need to address Positive Mental Health (2). This construct of Positive Mental Health was defined by WHO as the basis of well-being and effective functioning of people (3).

The professionals with PMH higher PMH are more successful in coping with risk situations such as an extreme and traumatic event, and therefore, they can exhibit fewer stress symptoms. Healthcare professionals in the emergency ambulance service run a higher risk of developing mental health disorders than the general population (4). Healthcare professionals face a series of stressors and mental health challenges due to the unique and demanding nature of their work. Some of the mean challenges include exposure to traumatic situations, rapid decision-making, irregular workloads and schedules, the risk of injuries and infectious diseases through contagion, interaction with patients and families in crisis situations, emotional exhaustion and difficulties in disconnecting. Also, in a study conducted by Søvold LE et al. highlighted there are several reasons why stress levels are high among healthcare professionals. These include a lack of physical or psychological safety, dealing with chronic care situations, experiencing moral conflicts and facing workplace related bullying (5).

Positive mental health (PMH) was first introduced by Marie Jahoda, defining it as a sense of physical, psychological, and social well-being (6). PMH is characterized as a state of feeling good and functioning effectively (6, 7). It describes the cognitive processes, emotional states and behaviors of individuals together in the way people are. PMH also contributes positively to people’s well-being; it is considered a benefit in relation to their aptitude for cognitive processes and interpersonal communication, as well as their capability to identify, comprehend, and interpret diverse situations, and if required, modify them (7–9). Similarly, PMH expresses the capacity to enjoy life, to promote autonomy and inclusion. PMH has been recognized as a fundamental element contributing to the positive functioning and psychological well-being of individuals, particularly in the context of leading a purposeful and meaningful life, with social engagement, personal achievement and in the development of skills and abilities through providing individuals with self-knowledge (9–11). Interest in investigating positive dimensions of health for example, there has been an improvement in aspects such as social inclusion, self-efficacy and the perceived ability to improve people’s health in recent years (9, 12). Assessing the PMH status of the general population, and also of specific groups, is important not only to understand which factors or variables in the individual’s environment influence and reinforce this construct, but also to design programmers to promote PMH in different contexts and populations. In this respect, the hospital environment serves as a reference point for both health professionals and the community to which they provide health care.

Orem and Vandiman pointed out that nurses, and health professionals in general, need to have good PMH skills (13). In addition, they should be able to detect the positive mental strengths of the people they care for, from a mental health point of view, even when their patients are experiencing an illness process. Thus, promoting health in work settings involves embracing a positive outlook, emphasizing a harmonious blend of preventive measures and initiatives aimed at enhancing the well-being of individuals and communities (14).

Interest in a positive perspective on mental health continues to grow and strengthen at the beginning of the 21st century (2, 13, 15). Other contributions that are recognized in the field of PMH are those presented by Lluch (16) when evaluating and operationalizing Jahoda’s PMH model. Lluch proposed a Multifactorial Model explaining PMH (MMPMH) based on six interrelated factors: Personal satisfaction (F1), Prosocial attitude (F2), Self-control (F3), Autonomy (F4), Problem solving and Self-Fulfilment (F5) and Interpersonal relationship skills (F6). The six factors as well as a description of each of them are shown in Table 1. To evaluate the Multifactorial Model of Positive Mental Health, the researcher developed the Positive Mental Health Questionnaire (PMHQ). Lluch’s PMHQ has been applied in various research studies and diverse contexts, including among individuals diagnosed with schizophrenia (17); chronic patients (18–20); caregivers of people with schizophrenia (21) and formal and informal caregivers of people with chronic illnesses (22, 23); mental health in-patient care professionals (24); university students (9, 25) and university professors (26).

**Table 1:** Multifactor model of positive mental health (16).

PMH factors	Definition
F1: Personal satisfaction	Self-concept/Self-esteem Satisfaction with personal life Optimistic outlook on the future
F2: Prosocial attitude	Active predisposition toward society Altruistic social attitude; attitude of helping/supporting others Acceptance of others and of differential social characteristics
F3: Self-control	Ability to cope with stress/situations of conflict Emotional balance/emotional control Tolerance of frustration, anxiety and stress
F4: Autonomy	Able to have one’s own standards Independence Self-regulation of one’s behavior Sense of personal security/self-confidence
F5: Problem-solving and self-actualization	Analytical capacity Ability to make decisions Flexibility/ability to adapt to change Attitude of continuous growth and personal development
F6: Interpersonal relationship skills	Ability to establish interpersonal relationships Empathy/ability to understand the feelings of others Ability to give emotional support Ability to establish and maintain close interpersonal relationships

PMH, Positive Mental Health.

On the other hand, the health-promoting model of salutogenesis is a conceptual model of human response to stress that takes the socio-cultural context and its influence on people’s health into consideration (27, 28). Antonovsky (29) identifies, defines, and describes the resources and factors that generate health at the individual and community level, through the Sense of Coherence (SOC) concept. SOC is defined as a global and non-specific orientation that portrays the Comprehensibility(C), Manageability (MA) and Meaningfulness (ME) of an individual’s perception of life as dimensions of health. A solid sense of coherence (SOC) allows individuals to have more self-confidence, giving them the ability to use the resources in their environment effectively. This empowerment in the use of resources promotes general well-being (30). The SOC is made up of three dimensions: comprehensibility (C) refers to the fact that life events follow a structured and understandable pattern. Manageability (MA) is related to the belief that one has control over life events. Finally, Meaningfulness (ME) implies the belief that life events, in addition to being valuable, provide personal satisfaction (31).

Individuals with a strong life orientation have been found to be healthier than those with a weak life orientation making this concept interesting for health and promotion (31). To assess SOC, Antonovsky constructed the Orientation to Life Questionnaire (OLQ-29). The 29-item instrument was designed to measure the three dimensions of health: comprehensibility, manageability, and meaningfulness. Years later, a shortened version, the 13-item SOC (OLQ-13), was validated (32). Research on the structure of the SOC scale has shown that rather than a unidimensional instrument for measuring health, the scale points toward a multidimensional construct (33–35).

The growing body of research on mental health within the healthcare profession highlights the need for additional evidence in this area, particularly with regard to Positive Mental Health (PMH) among healthcare professionals in the emergency ambulance service and how SOC may be a predictor variable of PMH levels. Health professionals working in the emergency ambulance service, care for people in highly vulnerable health situations and are involved in complex care processes (36). They are responsible for providing health care to the entire population in a critical situation or at potential risk at the individual and/or collective level. Due to the nature of their work, health professionals in the emergency ambulance service “are at a higher risk of developing mental health disorders than the general population” (37). According to Seyedjavadi et al., “about 74% of emergency health care professionals in Iran reported moderate levels of stress” (38). Preserving and improving the PMH of healthcare workers in the emergency ambulance service is crucial. This imperative extends beyond the well-being of healthcare professionals themselves; it encompasses the well-being of the communities they serve as well (39).

According to Foster et al., health in the work context enables individuals to cope with “their professional challenges, facilitating adaptation to stressful working conditions, management of emotions, development of coping strategies, improvement of well-being and personal growth” (40). For all the above reasons, the study of PMH and the relationship with SOC and demographic and occupational variables in health professionals in the emergency ambulance service can help us to understand the health predictor variables of professionals and, therefore, improve the quality of care they provide to individuals and communities. There is evidence regarding studies on levels of PMH and the dimensions of SOC in different populations (41, 42). However, no previous studies are known to address the predictive model of PMH in healthcare professionals. This is why we conducted this study. The objectives were, firstly, to identify the level of PMH and SOC in the study population. Evaluate PMH and its relationship with demographic and occupational variables and determine if SOC is a predictive variable for PMH in health professionals working in the emergency ambulance service.

## Materials and methods

### Participants

The population consisted of 493 emergency ambulance service healthcare professionals from eight health regions of Catalonia, Spain. The professionals were classified according to the professional category into health graduates and ambulance technicians. Emergency ambulance services are publicly managed, attached to the Department of Health of the Generalitat de Catalunya, Spain. They are specifically designed, staffed, and equipped for the emergency care of patients. This service is provided by a team of professionals, whose main objective is to respond to emergency ambulance service health and emergencies quickly, efficiently and with the highest level of quality, 24 h a day, 365 days a year. For the inclusion criteria of study participants, were considered all health professionals and special services personnel on the regular staff of the medical emergency system with experience equal to or greater than 1 year. Furthermore, they agreed to participate voluntarily after being informed about the study. Exclusion criteria for participants involved students and professionals undergoing training or updates who were not part of the regular staff of the medical emergency system and had less than 1 year of work experience. Professionals working in coordination centers handling telephone calls were also excluded, as it was deemed a task with less emotional impact. Additionally, all professionals on sick leave during the study period were excluded. A total of 406 healthcare professionals finally took part.

### Measures instruments

To understand the relationship between PMH and the demographic and occupational variables of the professionals and determine if SOC is a predictive variable for PMH, the following variables were analyzed (1): sociodemographic variables (*Age, biological sex, household members* and *dependent family members*) (2), occupational variables (*professional category, type of contract, job satisfaction* and *Sens of Collaboration with other institutions*) (3), PMHQ and (4) SOC. The following sections provide descriptions of the instruments employed.

### Positive mental health questionnaire

The PMH variable was assessed using the Positive Mental Health Questionnaire [PMHQ: (16)]. This questionnaire comprises 39 items Spanish version, which are unevenly distributed across the six factors that define the construct: F1-Personal Satisfaction (8 items), F2- Prosocial Attitude (5 items), F3- Self-control (5 items); F4- Autonomy (5 items); F5- Problem Solving and Self-actualization (9 items) and F6- Interpersonal Relationship Skills (7 items). The questionnaire provides a global score for PMH (sum of the items scores) as well as specific scores for each factor. The global PMH value ranges from 39 points (low PMH) to 156 points (high PMH). The minimum and maximum values for each factor are: 8–32 (factor F1), 5–20 (factor F2), 5–20 (factor F3), 5–20 (factor F4), 9–36 (factor F5) and 7–28 (factor F6) (Table 1). The PMHQ has been validated in different studies (43). The psychometric analyses of the original PMHQ conducted with a sample of nursing students were favorable (Lluch,1999). In this study, Cronbach’α on the global scale was 0.90, and by factors: F1 = 0.70; F2 = 0.60; F3 = 0.77; F4 = 0.66; F5 = 0.80; F6 = 0.64.

### Sense of coherence

The Sense of Coherence questionnaire (SOC-13) is a 13-item Spanish version based on the Orientation Life Questionnaire (OLQ) (44). The SOC was used to assess a global orientation to life, reflecting the ability of emergency ambulance service healthcare professionals to view life as comprehensible, manageable, and meaningful (29). As an example, comprehensibility was measured using items such as: “When you talk to people, do you have the feeling that they do not understand you?” (response alternatives: from “never have this feeling”- “always have this feeling”). Manageability was assessed using the item: “Do you have the feeling that you are being treated unfairly?” (from “very often” to “very seldom or never”). The item: “How often do you have the feeling that there’s little meaning in the things you do in your daily life?” (from “very often” to “very seldom or never”) gives an example of how meaningfulness was measured. A total sum was calculated, ranging from 13 to 91 points. Higher scores indicated stronger SOC. Cronbach’α values ranging from 0.72 to 0.89 for dimensions and 0.83 to 0.93 for the total SOC have been reported in previous studies. The internal consistency of the scale in the Spanish population is 0.80. Principal component analysis provided a multifactorial solution with a percentage of explained variance of 65.59%.

### Procedures

The participants were healthcare professionals working in the emergency ambulance service at eight bases in a health region of Catalonia, Spain. The care managers of each base participating in the study were contacted and a presentation of the project was scheduled at each one. It was approved by the management of the eight healthcare regions in Catalonia, Spain. The presentation included an explanation of the research objectives, the measurement instruments, informed consent and the procedure for distributing the data collection booklet in which the participants were informed of the voluntary nature of participation, anonymity and confidentiality of the data. The individuals participating in the study on a voluntary basis were healthy and did not have a professional or other relationship with the researcher. All healthcare professionals signed the informed consent form before participating in the study. The completed questionnaires did not contain any personal information that could identify the participants.

### Statistical analysis

The data were analyzed using SPSS 24.0 statistical software (IBM CORP., Armonk, NY). Forms with missing fields or invalid data were excluded from the analysis. A descriptive analysis was performed using statistics such as frequency, mean, and standard deviation, depending on the variable type. In order to analyze the existence of statistically significance differences in each of variables of the study (sociodemographic and occupational variables), bivariate analyses were carried out including Student’s T-Test, Analysis of Variance-ANOVA, and Pearson’s correlation. The reliability of the PMHQ was calculated using Cronbach’s alpha coefficient, with an *α* greater than 0.90 indicating acceptable internal consistency and reliability. A multivariate linear regression analysis was also performed to estimate the relationships between different variables. Researchers participating in this study considered *p* ≤ 0.05 as the confidence level (*p* value).

## Results

### Subjects’ characteristics

A total of 406 professionals from the emergency ambulance service healthcare system participated in the study. The majority of participants were men (66.7%) and the average age was 38.2 years (SD 7.5). The majority lived with their partner and/or family at the time of the study (83.9%) and had dependents family members (64.9%). Regarding the professional category, 50.5% of the sample studied were health graduate and 49.5% were ambulance technicians. A total of 90.2% of participants had a permanent contract. As for the SOC score, emergency ambulance service professionals reported a mean score of 69 (SD 10.7), with scores ranging from 13 to 91 points (range = 78). The sociodemographic and occupational characteristics of the study sample are shown in Table 2.

**Table 2:** Sociodemographic and occupational characteristics, sense of coherence variable on a global level and by factors.

Characteristics (*N* = 406)	*N*	Frequency %	Mean (SD)
Biological Sex (*n* = 403)			
Female	134	33.3	
Male	269	66.7	
Household members (*n* = 404)			
Living alone	65	16.1	
Living with partner/family	339	83.9	
Dependent family members (*n* = 396)			
Yes	257	64.9	
No	139	35.1	
Professional category (*n* = 404)			
Health graduate	204	50.5	
Ambulance technicians	200	49.5	
Position (*n* = 398)			
Permanent	359	90.2	
Temporary	39	9.8	
Job satisfaction (*n* = 393)			
Unsatisfied	23	5.9	
Satisfied	370	94.1	
Sense of collaboration with other institutions (*n* = 394)			
Non-collaborative	42	10.7	
Collaborative	352	89.3	
Age (*n* = 403)			**38.2 (7.5)**
SOC			
Comprehensibility (C)			**25.0 (4.95)**
Manageability (MA)			**21.0 (3.89)**
Meaningfulness (ME)			**23.0 (3.80)**
Total SOC			**69.0 (10.73)**

SOC, Sense of Coherence. The bold values refer to the Mean and Standard Deviation (SD).

### Descriptive statistics and relationship between level of PMH and sociodemographic, occupational and sense of coherence variables

The mean total PMH score of the study participants was 131.8 (SD 13.3), with minimum and maximum scores of 40 and 154 points, respectively (range = 114). The descriptive statistics of factors of the PMHQ are shown in Table 3.

**Table 3:** Descriptive Statistics of Factors of positive mental health questionnaire.

PMHQ	Rang	Min	Max	Mean	SD
F1: Personal satisfaction	24	8	32	28.0	3.3
F2: Prosocial attitude	15	5	20	17.1	2.0
F3: Self-control	14	6	20	16.4	2.6
F4: Autonomy	15	5	20	16.7	2.3
F5: Problem-solving and Self-actualization	27	9	36	30.7	4.1
F6: Interpersonal relationship skills	21	7	28	22.5	3.0
PMHQ total score (39–156 points)	114	40	154	**131.8**	**13.3**

PMHQ, Positive Mental Health Questionnaire; SD, Standard Deviation. The bold values refer to the Mean and Standard Deviation (SD).

Tables 4, 5 shows the relationship between PMH and the sociodemographic, occupational and sense of coherence variables. The correlation between PMH and biological sex revealed statistically significant differences in two factors: women obtained higher scores for autonomy (F4) (*p* = 0.016); in contrast, male scored higher in problem-solving abilities and self-fulfilment (F5) (*p* = 0.048).

**Table 4:** Association between levels of PMH in Factors 1, 2, 3 and 4 with demographic, occupational and SOC variables.

Characteristics (*N* = 406)	*N*	F1-Personal satisfaction		F2-Prosocial attitude		F3-Self-control		F4-Autonomy	
		Mean (SD)	*p*	Mean (SD)	*p*	Mean (SD)	*p*	Mean (SD)	*p*
Age^1^ (*n* = 403)	403	r = −0.099	**0.047**	r = −0.047	0.350	r = −0.055	0.273	r = −0.07	0.153
Biological Sex^2^ (*n* = 403)									
Female	134	27.6 (3.3)	0.303	16.9 (2.1)	0.674	16.7 (2.6)	0.498	16.9 (2.2)	0.016
Male	269	28.3 (3.3)		17.7 (1.9)		16.0 (2.4)		16.3 (2.5)	
Household members^2^ (*n* = 404)									
Living alone	65	27.2 (4.3)	**0.01**	17.1 (2.3)	0.716	16.4 (2.5)	0.333	16.8 (3.0)	0.133
Living with partner/family	339	28.2 (3.1)		17.2 (2.0)		16.5 (2.6)		16.7 (2.2)	
Dependent family members^2^ (*n* = 396)									
Yes	257	27.9 (3.4)	0.7672	17.1 (2.2)	0.319	16.4 (2.6)	0.830	16.5 (2.4)	0.098
No	139	28.2 (3.2)		17.2 (1.9)		16.5 (2.5)		17.1 (2.1)	
Professional category^2^ (*n* = 404)									
Health graduate	204	28.0 (3.1)	0.291	17.2 (2.0)	0.648	16.2 (2.4)	**0.021**	16.5 (2.2)	**0.020**
Ambulance technician	200	28.2 (3.6)		17.1 (2.2)		16.7 (2.8)		17.0 (2.4)	
Position^2^ (*n* = 398)									
Permanent	359	28.0 (3.4)	0.473	17.1 (2.1)	0.148	16.4 (2.6)	0.961	16.7 (2.4)	0.783
Temporary	39	28.4 (2.9)		17.6 (1.8)		16.4 (2.3)		16.8 (1.7)	
Job satisfaction^2^ (*n* = 393)									
Unsatisfied	23	25.0 (6.3)	**0.001**	15.6 (3.8)	**0.001**	15.3 (3.6)	**0.018**	16.3 (3.9)	0.252
Satisfied	370	28.3 (2.8)		17.3 (1.9)		16.6 (2.5)		16.8 (2.3)	
Collaboration with other institutions^2^ (*n* = 394)									
Non-collaborative	42	27.11 (4.6)	**0.022**	16.1 (2.9)	**0.001**	16.3 (2.7)	0.661	17.2 (2.9)	0.241
Collaborative	352	28.3 (3.1)		17.3 (1.9)		16.5 (2.6)		16.7 (2.3)	
SOC^1^	406								
Comprehensibility (C)		r = −0.458	**0.001**	r = −0.263	**0.001**	r = −0.416	**0.001**	r = −0.324	**0.001**
Manageability (MA)		r = −0.485	**0.001**	r = −0.322	**0.001**	r = −0.428	**0.001**	r = −0.268	**0.001**
Meaningfulness (ME)		r = −0.486	**0.001**	r = −0.519	**0.001**	r = −0.416	**0.001**	r = −0.314	**0.001**
Total SOC		r = −0.560	**0.001**	r = −0.423	**0.001**	r = −0.495	**0.001**	r = −0.359	**0.001**

PMH, Positive Mental Health; SOC, Sense of Coherence; SD, Standard deviation; *p*, level of significance ≤ 0.05; bold, Pearson’s correlation;1: *t* Student Fisher’s: 2.

**Table 5:** Association between levels of PMH in total score and Factors 5 and 6 with sociodemographic, occupational and SOC variables.

Characteristics (*N* = 406)	*N*	F5-Problem solving and Self-actualization		F6- Interpersonal Relationship Skills		PMHQ total score	
		Mean (SD)	*p*	Mean (SD)	*p*	Mean (SD)	*p*
Age^1^ (*n* = 403)	403	r = −0.114	**0.020**	r = −0.089	0.076	r = −0.112	**0.025**
Biological Sex^2^ (*n* = 403)							
Female	134	30.5 (4.5)	**0.048**	22.3 (3.0)	0.837	131.8 (14.1)	0.767
Male	269	31.1 (3.1)		23.0 (2.8)		131.9 (11.6)	
Household members^2^ (*n* = 404)							
Living alone	65	31.5 (4.1)	0.393	22.5 (3.2)	0.867	131.6 (15.8)	0.989
Living with partner/family	339	30.6 (4.1)		22.6 (2.9)		131.9 (12.8)	
Dependent family members^2^ (*n* = 396)							
Yes	257	30.4 (4.4)	0.158	22.5 (3.2)	0.167	131.1 (14.0)	0.344
No	139	31.2 (3.5)		22.6 (2.6)		133.0 (11.7)	
Professional category2 (*n* = 404)							
Health graduate	204	30.3 (3.9)	**0.020**	22.5 (2.8)	0.156	130.9 (11.8)	**0.021**
Ambulance technician	200	31.1 (4.4)		22.7 (3.2)		132.9 (14.4)	
Position2 (*n* = 398)							
Permanent	359	30.7 (4.2)	0.556	22.5 (3.0)	0.142	131.6 (13.6)	0.333
Temporary	39	31.1 (3.2)		23.2 (2.6)		133.7 (9.5)	
Job satisfaction2 (*n* = 393)							
Unsatisfied	23	28.7 (7.6)	**0.011**	20.7 (4.5)	**0.001**	121.6 (27.1)	**0.001**
Satisfied	370	30.9 (3.7)		22.7 (2.8)		132.8 (11.2)	
Collaboration with other institutions2 (*n* = 394)							
Non-collaborative	42	29.9 (5.3)	0.143	21.6 (4.2)	**0.017**	128.3 (19.8)	**0.039**
Collaborative	352	30.9 (3.9)		22.8 (2.7)		132.6 (11.7)	
SOC^1^	406						
Comprehensibility (C)		r = −0.365	**0.001**	r = −0.317	**0.001**	r = −0.484	**0.001**
Manageability (MA)		r = −0.348	**0.001**	r = −0.274	**0.001**	r = −0.477	**0.001**
Meaningfulness (ME)		r = −0.477	**0.001**	r = −0.472	**0.001**	r = −0.600	**0.001**
Total SOC		r = −0.465	**0.001**	r = −0.414	**0.001**	r = −0.610	**0.001**

PMH, Positive Mental Health; SOC, Sense of Coherence; SD, Standard deviation; *p*, level of significance ≤ 0.05; bold, Pearson’s correlation;1: t Student Fisher’s: 2.

Statistically significant differences were found between professional category and autonomy (F4), ambulance technicians showed more self-confidence and belief in their ability to exercise their own judgment than health graduates (*p* < 0.05). Statistically differences were observed in job satisfaction regarding PMH factors: Study participants who reported feeling totally satisfied in their job also showed greater satisfaction with their personal life and future prospects (F1) (*p* < 0.05); in their helpful prosocial attitude toward others (F2) (*p* < 0.05); in their ability to cope with stress and conflict situations (F3) (*p* < 0.05); in their ability in problem solving and self-actualization (F5) (*p* < 0.05); greater empathy and/or ability to understand the feelings of others, and their ability to establish interpersonal relationships (F6) (*p* < 0.05). Ambulance technicians also reported higher total PMH scores compared to health graduates (*p* < 0.05). Moreover, the participants reported that working collaboratively with other institutions brought them greater personal satisfaction (F1) (*p* = 0.008), and it was related to maintaining a helpful attitude toward others (F2) (*p* = 0.001) and to showing a greater ability to initiate and maintain interpersonal relationships (F6) (*p* = 0.001), as well as having a higher total PMH score (*p* < 0.05). The correlation between total SOC score and its dimensions with global PMH and the specific factors was low, but significant (*r* = −0.610). In the negative sense, the correlation indicated that the lower sense of coherence, the higher global level of PMH. When analyzing the relationship between the specific PMHQ factors and the three dimensions of SOC, it was observed that all factors/dimensions correlated negatively but statistically weak relationship.

### Multivariate analysis of factors associated with positive mental health

Predictors of positive mental health were obtained by a multivariate linear regression analysis. Positive Mental Health Questionnaire (PMHQ) and the six factors are presented in Table 6. It can be observed that F1 (*ß* = 0.327; *p* = 0.001); F2 (*ß* = 0.448; *p* = 0.001); F3 (*ß* = 0.234; *p* = 0.001); F4 (*ß* = 0.233; *p* = 0.001); F5 (*ß* = 0.392; *p* = 0.001); F6 (*ß* = 0.386; *p* = 0.001) and PMHQ total score (*ß* = 0.458; *p* = 0.001) showed a statistically significant and positive association with Meaningfulness (ME). In addition, Comprehensibility (C) was identified as a predictor for a greater personal satisfaction (F1), self-control (F3), autonomy (F4), interpersonal skills (F6) and PMHQ total score. In contrast, Manageability (MA) was not found as a predictor in any of the PMHQ variables in the multivariate models. Regarding the occupational variables, on one hand being a technician was identified as a predictor of greater positive mental health and better problem solving and self-actualization (F5).

**Table 6:** Multivariate regression model for Factors and total Positive Mental Health with sociodemographic, occupational and Sense of Coherence variables.

Determinants	F1-Personal satisfaction		F2-Prosocial attitude		F3-Self-control		F4-Autonomy		F5-Problem solving and Self-actualization		F6- Interpersonal Relationship Skills		PMHQ total score	
	*β*	*p*	*β*	*p*	*β*	*p*	*β*	*p*	*β*	*p*	*β*	*p*	*β*	*p*
Sociodemographic variables														
Age	−0.029	0.535	0.015	0.768	0.021	0.683	0.026	0.635	−0.022	0.665	−0.023	0.654	−0.008	0.853
Biological Sex (Male/Female)	−0.132	**0.004**	0.131	**0.007**	−0.117	**0.019**	−0.111	**0.033**	0.039	0.427	0.084	0.091	−0.025	0.571
Household members (Alone/Others)	0.088	0.051	−0.016	0.732	0.009	0.858	−0.013	0.800	−0.067	0.175	−0.008	0.869	−0.004	0.918
Dependent family members (Yes/No)	0.090	0.061	0.027	0.601	0.069	0.188	0.140	**0.011**	0.092	0.081	−0.004	0.946	0.096	**0.039**
Occupational variables														
Professional Category (Graduate/Technician)	0.009	0.845	0.045	0.361	0.091	0.070	0.092	0.082	0.107	**0.34**	0.078	0.125	0.097	**0.028**
Position (Permanent/Temporary)	0.009	0.837	0.074	0.129	−0.002	0.976	0.014	0.786	0.006	0.898	0.093	0.063	0.040	0.361
Job satisfaction (Yes/No)	0.066	0.140	0.059	0.213	−0.030	0.543	−0.026	0.615	0.015	0.759	0.049	0.322	0.031	0.467
Collaboration with other institutions^2^ (No/Yes)	0.043	0.335	0.094	0.050	0.011	0.819	−0.106	0.**041**	−0.001	0.990	0.094	0.056	0.030	0.488
SOC variables														
Meaningfulness (ME)	0.327	**0.001**	0.448	**0.001**	0.234	**0.001**	0.233	**0.001**	0.392	**0.001**	0.386	**0.001**	0.458	**0.001**
Comprehensibility (C)	0.222	**0.001**	−0.009	0.881	0.246	**0.001**	0.247	**0.001**	0.156	0.015	0.183	**0.004**	0.242	**0.001**
Manageability (MA)	0.112	0.064	0.045	0.483	0.102	0.121	−0.019	0.786	−0.013	0.849	−0.118	0.075	0.021	0.711
*R* ^2^	**38.4%**		**29.8%**		**26.3%**		**18.6%**		**25.6%**		**25.7%**		**43.1%**	

PMH, Positive Mental Health; SOC, Sense of Coherence; *R*^2^, % explained variance; β, Standardized Beta; *p*, β p-level of significance ≤ 0.05; bold, Pearson’s correlation;1: *t* Student Fisher’s: 2.

**Figure 1:**
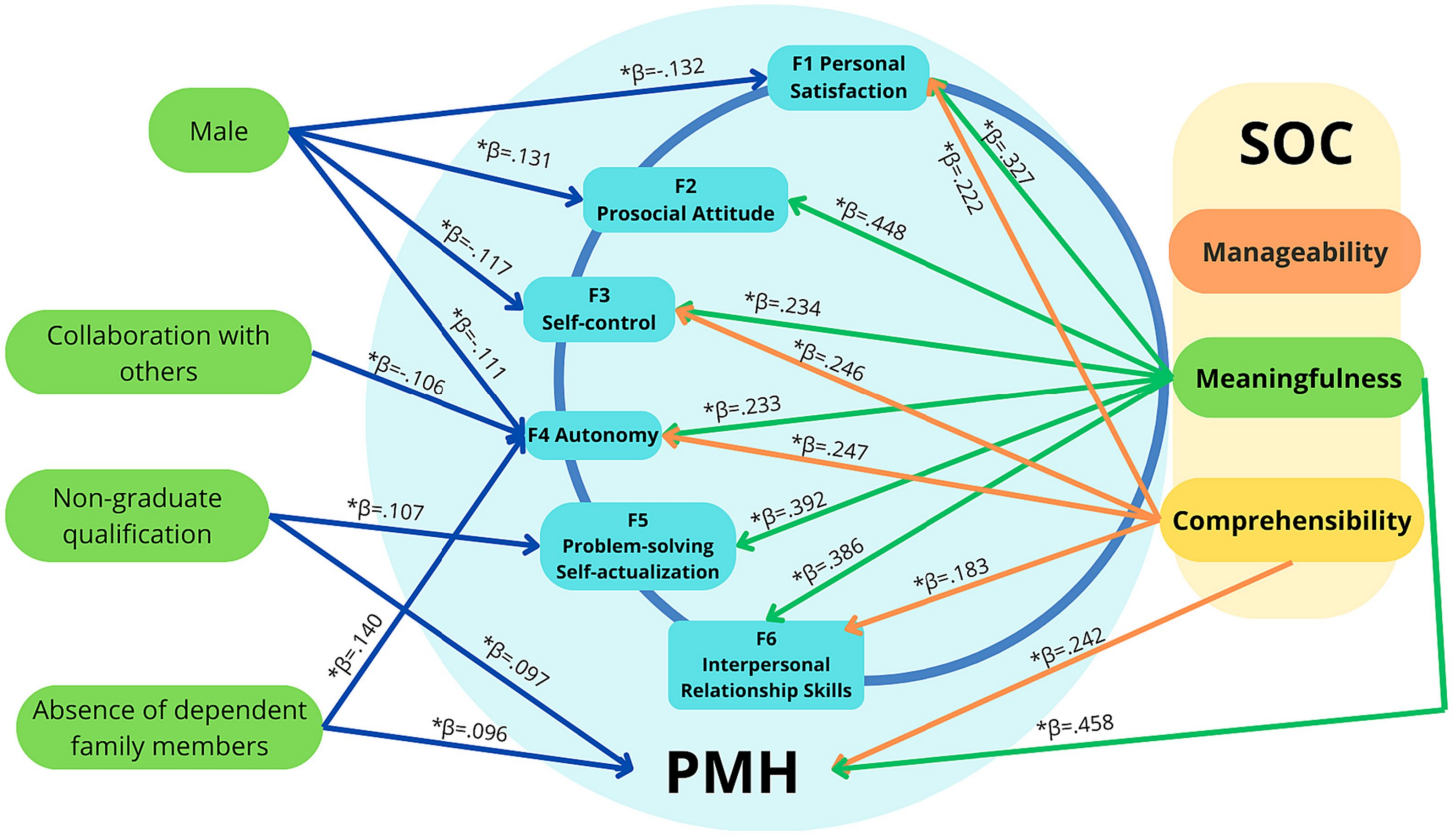
Predictive PMH modeling in health professionals. β: Standardized Beta, * β *p*-level of significance ≤0.05.

On the other hand, having a greater sense of collaboration was identified as a predictor of greater autonomy (F4). Regarding the sociodemographic variables, not having dependent family members was identified as a better autonomy predictor (F4) and greater positive mental health score overall. Moreover, being a male was a predictor for a greater prosocial attitude (F2), but also for a lower personal satisfaction (F1), self-control (F3) and autonomy (F4). According to the predictive model of the PMH, 43.1% of positive mental health in health professionals is explained by higher ME and C values of the SOC, the absence of dependent family members and having a non-graduate background. The predictive PMH model in health professionals is represented in Figure 1.

## Discussion

Professionals working in the emergency ambulance service healthcare system often encounter the traumatic experiences of the individuals they attend to. Consequently, they are more susceptible to experiencing mental health issues compared to professionals in other healthcare services. This has the potential to influence the quality of patient care, professional relationships with colleagues, and contribute to the development of more severe mental health conditions. Interest in PMH and SOC as predictors of good health in work environments has generated much research in the last 10 years. To the best of our knowledge, there are few studies about PMH and the predictor variables in healthcare emergency workers. For this reason, this study aimed to evaluate PMH and its relationship with demographic and occupational variables and determine if SOC is a predictive variable for PMH in this population. According to the predictive model on positive mental health proposed in our study, the SOC constitutes a predictor of the PMH of healthcare workers.

In our research, PMH levels were similar to those of other study samples, as in the study conducted by Albacar-Riobóo et al. (21) in a population of people diagnosed with schizophrenia, nursing students (16), pregnant women (45), adults with chronic diseases, both mental health disorders and physical health conditions (46, 47) or even in the caregivers’ informal reports of patients with chronic diseases (48, 49).

On the other hand, and related to the age of the participants in our study, we observed significant differences between PMH levels and age, results that align with the study by Lluch-Canut et al. (18), where age is also identified as a mediator of PMH. Other studies have also reported differences in PMH based on age and occupational variables (50, 51). It is crucial to continue considering this variable in future research to introduce specific interventions in PMH at earlier ages, as the overall level of PMH tends to decrease in old age (20).

When examining the relationship between biological sex and PMH, women scored higher on average in the F4-Autonomy factor than men. On the other hand, men scored higher in the F5-Problem Solving and Self-Actualization factor. These findings are consistent with studies conducted by Lluch-Canut et al. (18) and Jeyagurunathan et al. (52). These results can guide the design of interventions programs, taking into account the biological sex variable and the PMH factors that need improvement. Regarding the household members variable, living with family and/or partner was correlated with a significantly higher score in the F1-Personal satisfaction factor. In the same line, several studies have found that people who live as a couple show greater satisfaction and well-being (20, 50, 53), results that align with our study. In this line, and related to job satisfaction, 94% of the professional’s expressed satisfaction with their work, a result similar to that obtained in another study of healthcare professionals working in hospitals units in hospital units (24).

On the other hand, a noteworthy result of this study is the relationship found between PMH levels and the variable collaboration with other institutions, where significant differences were observed in the factors F1-Personal satisfaction, F2-Prosocial attitude, and F6-Interpersonal relationship skills. Collaborative work is considered a crucial factor for successfully implementing a Positive Mental Health (PMH) in the workplace (42). The knowledge and skills of various health professionals, such as professionals working in the emergency ambulance service, play an important role in the development of positive mental health in the professional context.

As for the SOC variable, according to Ericksson and Lindstrom (54), the variable Sense of Coherence (SOC) “has been empirically related to well-being” and quality of life, demonstrating to be “a protective factor against stressors” derived “from the work environment. In this line, other studies have linked low levels of SOC to health issues such as burnout or depression (41), and high levels of SOC to self-esteem and optimism (42). These results are a significant finding because they align with our study and with the proposed predictive model of PMH. Several studies reveal the relationship between positive mental health and SOC. In research examining the importance of social capital and SOC in relation to the mental health of refugees, findings indicated a positive association between SOC and the mental health of refugees (55). According to Penachiotti et al. (56) in their article “Sense of Coherence and social support as predictors of mental health during the COVID-19 pandemic,” the results revealed that SOC acts a predictive factor for the mental health of individuals in Brazil. This study aligns with our research, although there is still limited investigation into the predictive model of PMH, where SOC can act as a predictor. Further studies are needed to strengthen this finding.

According to our research, fostering the development of SOC can contribute to improve mental health. This result aligns with research on SOC and positive feedback to promote personal growth and flourishing (57). In another study conducted with healthcare workers in Ecuador during the early phase of the COVID-19 pandemic, there was a positive relationship between SOC and work engagement, and a negative relationship with psychological distress (58). Another study demonstrated that caregivers of people with dementia showed a positive correlation between their SOC and resilience, as well as their sense of competence and health-related quality of life (59). According to Veronese and Pepe (60), the research delved into whether the SOC functions as a factor influencing positive psychological well-being among humanitarian workers directly exposed to the rigors of war. Individuals without a particular professional background seemed to safeguard their mental health by adopting a SOC. Once again, these findings align with the resutlts of our study, indicating that SOC can act as a predictive variable for PMH in the workplace.

According to the PMH predictive model of this study, positive mental health in health professionals is explained by higher values of Meaningfulness (ME) and Comprehensibility (C) in the SOC variable. Numerous studies investigating the SOC in the workplace have shown that individuals with a strong SOC experienced fewer stress symptoms and demonstrated more effective coping mechanisms in response to work-related stress (61). In another study conducted by Kinman and Jones (62) similar results were obtained; employees with a strong SOC tended to have better physical and psychological health than those with a weaker SOC. Other longitudinal studies have demonstrated that a high level of SOC can reduce the risk of developing burnout symptoms 10 years later (63) and that SOC can protect against the negative effects of organizational change on mental health (64). These findings align with our study, and as more evidence accumulates, the scientific foundation supporting the relationship between SOC and PMH is strengthened. This is essential for establishing robust theories and grounding effective interventions in the workplace. Conducting additional studies can help identify the underlying causal mechanisms in the relationship between SOC and PMH. Understanding these mechanisms will contributes to developing more specific and effective interventions. Van der Colff and Rothmann (65) reported that “SOC is not only related to lower levels of burnout but also to higher levels of positive outcomes, such as work engagement.” Muller and Rothmann (66), conducted a content analysis of written responses from 600 employees, showing that the perception of helpful factors differed from limiting factors depending on employees’ SOC levels. Individuals with a high SOC may be more capable of positively changing the dynamics of their work environment, as indicated by a three-year longitudinal follow-up study (33). Consistent with our study, other research has indicated that elevated levels of SOC in healthcare professionals correlate with better health, increased work engagement, and a reduction in conflicts between work and family responsibilities (67). Midwives have shown a negative correlation between their SOC and stress levels, suggesting that a higher SOC is associated with lower stress in this professional group (68). López-Martínez et al. (69), suggest that SOC is a factor favoring the mental health of individual caregivers, as it relates to quality of life and protects against anxiety, depression, and the perceived workload burden. SOC has also been linked to the prevention of post-traumatic stress experienced by healthcare professionals (70). Consequently, these results agree with our study, where SOC has a positive impact on coping with workplace stress and serves as a predictor of PMH (61, 62, 71, 72). Longitudinal studies exploring the health promotion impact of linking SOC levels to exposure to adverse working conditions have demonstrated connections between SOC levels and psychosocial factors in the workplace (73). However, few studies have analyzed the predictive power of the SOC variable on PMH levels in professionals working in the emergency ambulance service. Our PMH predictive model in health professionals can contribute to confirming that SOC is a reliable predictor of PMH. Specific interventions focused on strengthening this variable could be designed, thereby enhancing the PMH of healthcare professionals.

### Limitations and practical implication

The limitations of this study include the composition of the participants, which is not representative of the population of Spain, although it is representative of the emergency medical services in a Catalonia. Replicating this study in other populations that are representative in similar professional contexts is needed to replicate the predictive PMH model in health professionals, produced in this study. Rigorous longitudinal designs should be employed to assess the consistency of results at different time points and understand whether the predictor variable (SOC) influences PMH over time. Another limitation of this study is that the gender perspective has not been examined in relation to the variables under study.

This study provides the first evidence of predictive PMH model in emergency healthcare professionals. Healthcare workers with high SOC scores had higher PMH, greater confidence in their decision-making abilities, increased personal satisfaction and demonstrated a greater ability to cope with challenges in their workplace. Although they are a different concept, Psychological First Aid (PFA) can act as preventive measure against stress or depression in individuals after exposure to stressful events. However, PFA functions as illness prevention, while SOC, on the contrary, promotes health by enhancing PMH. More studies are needed to provide evidence about that question, and it is undoubtedly a future research direction, given the impact it can have on the health on the health workers. This information will lay the groundwork for further investigations, including studies on the effectiveness of interventions that nay inform policies and practices in the future.

## Conclusion

Healthcare workers in the emergency ambulance service had high PMH. Meaningfulness (ME) and Comprehensibility (C), subscales of the SOC, were identified as model predictors of greater PMH, showing higher scores in most of PMH factors. To enhance SOC as a mental health promotion measure, resilience programs should be implemented to help professionals develop skills to face and overcome adverse situations. Educating in stress management thought networks are key elements to strengthen SOC. Likewise, developing programs that enhance decision-making skills would improve levels of ME and C in SOC. Managers in emergency medical services play a key role in transforming healthcare work environments to promote positive outcomes in the mental health of their healthcare workers. This could be part of an effective promotion program aimed at sustaining long-term health in healthcare institutions.

## Data availability statement

The original contributions presented in the study are included in the article/supplementary material, further inquiries can be directed to the corresponding author.

## Ethics statement

The studies involving humans were approved by Clinical Research Ethics Committee on Clinical Research of the Emergency Medical System (EMS). The studies were conducted in accordance with the local legislation and institutional requirements. The participants provided their written informed consent to participate in this study. The animal study was approved by Clinical Research Ethics Committee on Clinical Research of the Emergency Medical System (EMS). The study was conducted in accordance with the local legislation and institutional requirements. Written informed consent was obtained from the individual(s) for the publication of any potentially identifiable images or data included in this article.

## Author contributions

SM-J: Conceptualization, Methodology, Writing – original draft. GR-G: Formal analysis, Methodology, Supervision, Writing – review & editing. MR-V-L: Formal analysis, Methodology, Writing – review & editing. DC-L: Conceptualization, Supervision, Writing – review & editing. AM-P: Investigation, Methodology, Supervision, Writing – review & editing. ML-C: Investigation, Methodology, Supervision, Writing – review & editing. DJ-C: Conceptualization, Methodology, Supervision, Writing – review & editing.

## References

[1] Teixeira S Coelho J Sequeira C Lluch I Canut MT Ferré-Grau C. The effectiveness of positive mental health programs in adults: a systematic review. Health Soc Care Community. (2019) 27:1126–34. doi: 10.1111/hsc.12776, PMID: 31144395

[2] World Health Organization. The European mental health action plan 2013–2020. Copenhagen: WHO Regional Office for Europe (2015).

[3] World Health Organization. *Quinta Conferencia Internacional de Promoción de la Salud. Promoción de la salud: hacia una mayor equidad*. Ciudad de México (2000). Available at: http://www.who.int/healthpromotion/conferences/previous/mexico/en/hpr_mexico_report_sp.pdf.

[4] Sterud T Ekeberg Ø Hem E. Health status in the ambulance services: a systematic review. BMC Health Serv Res. (2006) 6:82. doi: 10.1186/1472-6963-6-82, PMID: 16817949 PMC1559607

[5] Søvold LE Naslund JA Kousoulis AA Saxena S Qoronfleh MW Grobler C. Prioritizing the mental health and well-being of healthcare workers: an urgent global public health priority. Front Public Health. (2021) 9:679397. doi: 10.3389/fpubh.2021.679397, PMID: 34026720 PMC8137852

[6] Jahoda M. Current concepts of positive mental health. New York: Basic Books (1958).

[7] Canadian Institute for Health Information. Improving the health of Canadians: Exploring positive mental health. Ottawa, Ontario, Canada: Canadian Institute for Health Information (2009).

[8] Lehtinen V Sholman B Kovess-Masfety V. Level of positive mental health in the European Union: results from the Eurobarometer 2002 survey. Clin Pract Epidemiol Ment Health. (2005) 1:9. doi: 10.1186/1745-0179-1-9 16042763 PMC1188064

[9] Lluch-Canut MT. Empirical evaluation of a conceptual model of positive mental health. Salud Ment. (2002) 25:42–55.

[10] Lluch-Canut MT. Development and psychometric analysis of a positive mental health questionnaire. Behav Psychol. (2003) 11:61–78.

[11] Roldán-Merino J Lluch-Canut MT Casas I Sanromà-Ortíz M Ferré-Grau C Sequeira C. Reliability and validity of the positive mental health questionnaire in a sample of Spanish university students. J Psychiatr Ment Health Nurs. (2017) 24:123–33. doi: 10.1111/jpm.12358, PMID: 28150373

[12] Ryff CD. Psychological well-being revisited: advances in the science and practice of eudaimonia. Psychother Psychosom. (2014) 83:10–28. doi: 10.1159/000353263, PMID: 24281296 PMC4241300

[13] Orem DE Vardiman EM. Orem's nursing theory and positive mental health: practical considerations. Nurs Sci Q. (1995) 8:165–73. doi: 10.1177/089431849500800407, PMID: 8684725

[14] Cassetti V Powell K Barnes A Sanders T. A systematic scoping review of asset-based approaches to promote health in communities: development of a framework. Glob Health Promot. (2020) 27:15–23. doi: 10.1177/1757975919848925, PMID: 31319777

[15] World Health Organization. Health 2020: A European policy framework supporting action across government and society for health and well-being. Copenhagen: WHO Regional Office for Europe (2012).

[16] Lluch MT. *Construcción de una escala para evaluar la salud mental positiva*. PhD Thesis. Faculty of Psychology, University of Barcelona (1999).

[17] Miguel-Ruiz MD. *Valoración de la Salud Mental Positiva y de los Requisitos de Autocuidado, en pacientes hospitalizados diagnosticados de Esquizofrenia, según la Teoría de Enfermería de Dorothea Orem*. PhD Thesis. University of Barcelona (2014).

[18] Lluch-Canut T Puig-Llobet M Sánchez-Ortega A Roldán-Merino J Ferré-Grau C Positive Mental Health Research Group. Assessing positive mental health in people with chronic physical health problems: correlations with socio-demographic variables and physical health status. BMC Public Health. (2013) 13:928. doi: 10.1186/1471-2458-13-928, PMID: 24093443 PMC3853147

[19] Sánchez-Ortega MA. *Efectividad de un programa de intervención psicosocial enfermera para potenciar la agencia de autocuidado y la salud mental positiva en personas con problemas crónicos de salud*. PhD Thesis. University of Barcelona (2015).

[20] Puig Llobet M Sánchez Ortega M Lluch-Canut M Moreno-Arroyo M Hidalgo Blanco MÀ Roldán-Merino J. Positive mental health and self-Care in Patients with chronic physical health problems: implications for evidence-based practice. Worldviews Evid-Based Nurs. (2020) 17:293–300. doi: 10.1111/wvn.12453, PMID: 32762130

[21] Albacar-Ribóo N Lleixá-Fortuño M Lluch Canut M Sequeira C Carvalho JC Roldán Merino JF. Propiedades psicométricas de la versión autoadministrada de la “Escala de Requisitos de Autocuidado” entre los cuidadores de enfermos mentales. Rev Port Enferm Saúde Ment. (2015) 13:50–60.

[22] Ferré-Grau C Raigal-Aran L Lorca-Cabrera J Ferré-Bergadá M Lleixà-Fortuño M Lluch-Canut MT. A multi-Centre, randomized, 3-month study to evaluate the efficacy of a smartphone app to increase caregiver’s positive mental health. BMC Public Health. (2019) 19:888. doi: 10.1186/s12889-019-7264-5, PMID: 31277623 PMC6612114

[23] Ferré-Grau C Raigal-Aran L Lorca-Cabrera J Lluch-Canut MT Ferré-Bergada M Lleixà-Fortuño M. A mobile app-based intervention program for nonprofessional caregivers to promote positive mental health: randomized controlled trial. JMIR Mhealth Uhealth. (2021) 9:e21708. doi: 10.2196/21708, PMID: 33480852 PMC7864775

[24] Mantas S Juvinyà D Bertran C Roldán J Sequeira C Lluch T. Evaluación de la Salud Mental Positiva y sentido de coherencia en profesionales de la salud mental. Rev Port Enferm Saude Ment. (2015) 13:34–42.

[25] Sequeira C Carvalho JC Gonçalves A Nogueira MJ Lluch-Canut T Roldán-Merino J. Levels of positive mental health in Portuguese and Spanish nursing students. J Am Psychiatr Nurses Assoc. (2020) 26:483–92. doi: 10.1177/1078390319851569, PMID: 31122109

[26] Hurtado B Moreno C Casas I Lluch MT Lleixà M Farrés M. Positive mental health and prevalence of psychological ill-being in university nursing professors in Catalonia, Spain. J Psychosoc Nurs Ment Health Serv. (2017) 55:38–48. doi: 10.3928/02793695-20170619-06, PMID: 28671240

[27] Hosrburg ME. Salutogenesis: origins and health of sense of coherence In: Hill Rice V, editor. Handbook of stress: Coping and health: Implications for nursing research theory and Practise. California: Sage Publications (2000). 175–94.

[28] Lindström B Eriksson M. Salutogenesis. J Epidemiol Community Health. (2005) 59:440–2. doi: 10.1136/jech.2005.034777, PMID: 15911636 PMC1757059

[29] Antonovsky A. Health promoting factors at work: the sense of coherence In: Kalimo R El-Batawi MA Cooper CL, editors. Psychosocial factors at work and their relation to health. Geneva, Switzerland: World Health Organization (1987). 153–67.

[30] Antonovsky A. The salutogenic model as a theory to guide health promotion. Health Promot Int. (1996) 11:11–8. doi: 10.1093/heapro/11.1.11

[31] Eriksson M Lindström B. Antonovsky’s sense of coherence scale and the relation with health - a systematic review. J Epidemiol Community Health. (2006) 60:376–81. doi: 10.1136/jech.2005.041616, PMID: 16614325 PMC2563977

[32] Antonovsky A. The structure and properties of the sense of coherence scale. Soc Sci Med. (1993) 36:725–33. doi: 10.1016/0277-9536(93)90033-Z 8480217

[33] Feldt T Kinnunen U Mauno S. A mediational model of sense of coherence in the work context: a 1–year follow-up study. J Organ Behav. (2000) 21:461–76. doi: 10.1002/(SICI)1099-1379(200006)21:4<461::AID-JOB11>3.0.CO;2-T

[34] Feldt T Kokko K Kinnunen U Pulkkinen L. The role of family background, school success, and career orientation in the development of sense of coherence. Eur Psychol. (2005) 10:298–308. doi: 10.1027/1016-9040.10.4.298

[35] Feldt T Lintula H Suominen S Koskenvuo M Vahtera J Kivimäki M. Structural validity and temporal stability of the 13-item sense of coherence scale: prospective evidence from the population-based HeSSup study. Qual Life Res. (2007) 16:483–93. doi: 10.1007/s11136-006-9130-z, PMID: 17091360

[36] Shakespeare-Finch J Daley E. Workplace belongingness, distress, and resilience in emergency service workers. Psychol Trauma. (2017) 9:32–5. doi: 10.1037/tra0000108, PMID: 27243572

[37] Hummel S Oetjen N Du J Posenato E Resende de Almeida RM Losada R. Mental health among medical professionals during the COVID-19 pandemic in eight European countries: cross-sectional survey study. J Med Internet Res. (2021) 23:e24983. doi: 10.2196/24983, PMID: 33411670 PMC7817254

[38] Seyedjavadi M Samadi N Mohammadi R Osmani A Bakhtiari Kohsareh F Seyedjavadi M. Assessment of stress in medical emergency staff in Ardabil Province, Iran. Qom Univ Med Sci J. (2014) 7:41–5.

[39] Halpern J Gurevich M Schwartz B Brazeau P. What makes an incident critical for ambulance workers? Emotional outcomes and implications for intervention. Work Stress. (2009) 23:173–89. doi: 10.1080/02678370903057317

[40] Foster K Cuzzillo C Furness T. Strengthening mental health nurses' resilience through a workplace resilience programme: a qualitative inquiry. J Psychiatr Ment Health Nurs. (2018) 25:338–48. doi: 10.1111/jpm.12467, PMID: 29920873

[41] Basińska MA Andruszkiewicz A Grabowska M. Nurses' sense of coherence and their work related patterns of behaviour. Int J Occup Med Environ Health. (2011) 24:256–66. doi: 10.2478/S13382-011-0031-1, PMID: 21833694

[42] Garrosa E Rainho C Moreno-Jiménez B Monteiro MJ. The relationship between job stressors, hardy personality, coping resources and burnout in a sample of nurses: a correlational study at two time points. Int J Nurs Stud. (2010) 47:205–15. doi: 10.1016/j.ijnurstu.2009.05.014, PMID: 19596324

[43] Sequeira C Carvalho JC Sampaio F Sá L Lluch-Canut T Roldán-Merino J. Evaluation of the psychometric properties of the positive mental health questionnaire in Portuguese higher education students. Rev Port Enferm Saude Ment. (2014) 11:45–53.

[44] Virués-Ortega J Martínez-Martín P Del Barrio JL Lozano LM. Crosscultural validation of Antonovsky’s Sense of Coherence Scale (OLQ-13) in Spanish elders aged 70 years or more. Med Clin (Barc). (2007) 128:486–92. doi: 10.1157/13100935 17419910

[45] Monterrosa-Castro Á Romero-Martínez S Monterrosa-Blanco A. Positive maternal mental health in pregnant women and its association with obstetric and psychosocial factors. BMC Public Health. (2023) 23:1013. doi: 10.1186/s12889-023-15904-4, PMID: 37254059 PMC10227798

[46] Broncano-Bolzoni M González-Carrasco M Juvinyà-Canal D Lluch-Canut M. The mental health of patients with psychotic disorder from a positive, multidimensional and recovery perspective. Front Psychol. (2022) 13:857598. doi: 10.3389/fpsyg.2022.857598, PMID: 35859819 PMC9290860

[47] Sánchez-Ortega MA Lluch-Canut MT Roldán-Merino J Agüera Z Hidalgo-Blanco MA Moreno-Poyato AR. Nursing intervention to improve positive mental health and self-care skills in people with chronic physical health conditions. Int J Environ Res Public Health. (2022) 20:528. doi: 10.3390/ijerph20010528, PMID: 36612849 PMC9819309

[48] Ferré-Bergadà M Valls A Raigal-Aran L Lorca-Cabrera J Albacar-Riobóo N Lluch-Canut T. A method to determine a personalized set of online exercises for improving the positive mental health of a caregiver of a chronically ill patient. BMC Med Inform Decis Mak. (2021) 21:74. doi: 10.1186/s12911-021-01445-6, PMID: 33632207 PMC7905974

[49] Tinoco-Camarena JM Puig-Llobet M Lluch-Canut MT Roldan-Merino J Moreno-Arroyo MC Moreno-Poyato A. Effectiveness of the online "dialogue circles" nursing intervention to increase positive mental health and reduce the burden of caregivers of patients with complex chronic conditions. Randomized clinical trial. Int J Environ Res Public Health. (2022) 20:644. doi: 10.3390/ijerph20010644, PMID: 36612964 PMC9819240

[50] Vaingankar JA Subramaniam M Chong SA Abdin E Orlando Edelen M Picco L. The positive mental health instrument: development and validation of a culturally relevant scale in a multi-ethnic Asian population. Health Qual Life Outcomes. (2011) 9:92. doi: 10.1186/1477-7525-9-92, PMID: 22040157 PMC3229450

[51] Cheng HL Wang C McDermott RC Kridel M Rislin JL. Self-stigma, mental health literacy, and attitudes toward seeking psychological help. J Couns Dev. (2018) 96:64–74. doi: 10.1002/jcad.12178

[52] Jeyagurunathan A Sagayadevan V Abdin E Zhang Y Chang S Shafie S. Psychological status and quality of life among primary caregivers of individuals with mental illness: a hospital based study. Health Qual Life Outcomes. (2017) 15:106. doi: 10.1186/s12955-017-0676-y, PMID: 28526049 PMC5438522

[53] European Commission. Mental health. Special Eurobarometer 345/wave 73.2. Brussels: European Commission (2011).

[54] Teke C Baysan AL. The validity and reliability of positive mental health scale. Turk Psikiyatri Derg. (2018) 19:1–28. doi: 10.5455/apd.284116

[55] van Sint Fiet A de la Rie S van der Aa N Bloemen E Wind T. The relevance of social capital and sense of coherence for mental health of refugees. SSM Popul Health. (2022) 20:101267. doi: 10.1016/j.ssmph.2022.101267, PMID: 36281249 PMC9587331

[56] Penachiotti FDF Yamaguchi MU Mana A Sagy S Grossi-Milani R. Sense of coherence and social support as predictors of mental health during COVID-19 pandemic. Rev Bras Enferm. (2023) 76:e20220468. doi: 10.1590/0034-7167-2022-0468, PMID: 37556675 PMC10405388

[57] Tušl M de Bloom J Bauer GF. Sense of coherence, off-job crafting, and mental well-being: a path of positive health development. Health Promot Int. (2022) 37:159. doi: 10.1093/heapro/daac159, PMID: 36440899 PMC9703811

[58] Gómez-Salgado J Arias-Ulloa CA Ortega-Moreno M García-Iglesias JJ Escobar-Segovia K Ruiz-Frutos C. Sense of coherence in healthcare workers during the COVID-19 pandemic in Ecuador: association with work engagement, work environment and psychological distress factors. Int J Public Health. (2022) 67:1605428. doi: 10.3389/ijph.2022.1605428, PMID: 36545403 PMC9760665

[59] Stansfeld J Orrell M Vernooij-Dassen M Wenborn J. Sense of coherence in family caregivers of people living with dementia: a mixed-methods psychometric evaluation. Health Qual Life Outcomes. (2019) 17:44. doi: 10.1186/s12955-019-1114-0, PMID: 30866961 PMC6417216

[60] Veronese G Pepe A. Sense of coherence as a determinant of psychological well-being across professional groups of aid workers exposed to war trauma. J Interpers Violence. (2017) 32:1899–920. doi: 10.1177/0886260515590125, PMID: 26088898

[61] Albertsen K Nielsen ML Borg V. The Danish psychosocial work environment and symptoms of stress: the main, mediating, and moderating role of sense of coherence. Work Stress. (2001) 15:241–53. doi: 10.1080/02678370110066562

[62] Kinman G Jones F. A life beyond work? Job demands, work-life balance, and wellbeing in UK academics. J Hum Behav Soc Environ. (2008) 17:41–60. doi: 10.1080/10911350802165478

[63] Kalimo R Pahkin K Mutanen P Topipinen-Tanner S. Staying well or burning out at work: work characteristics and personal resources as long-term predictors. Work Stress. (2003) 17:109–22. doi: 10.1080/0267837031000149919

[64] Pahkin K Väänänen A Koskinen A Bergbom B Kouvonen A. Organizational change and employees' mental health: the protective role of sense of coherence. J Occup Environ Med. (2011) 53:118–23. doi: 10.1097/JOM.0b013e318206f0cb, PMID: 21270657

[65] Van der Colff JJ Rothmann S. Occupational stress, sense of coherence, coping, burnout and work engagement of registered nurses in South Africa. SA J Ind Psychol. (2009) 35:423. doi: 10.4102/sajip.v35i1.423

[66] Muller Y Rothmann S. Sense of coherence and employees’ perceptions of helping and restraining factors in an organisation. SA J Ind Psychol. (2009) 35:731. doi: 10.4102/sajip.v35i1.731

[67] Malagón-Aguilera MC Suñer-Soler R Bonmatí-Tomás A Bosch-Farré C Gelabert-Vilella S Juvinyà-Canal D. Relationship between sense of coherence, health and work engagement among nurses. J Nurs Manag. (2019) 27:1620–30. doi: 10.1111/jonm.12848, PMID: 31444895

[68] Gebriné KÉ Lampek K Sárváry A Sárváry A Takács P Zrínyi M. Impact of sense of coherence and work values perception on stress and self-reported health of midwives. Midwifery. (2019) 77:9–15. doi: 10.1016/j.midw.2019.06.006, PMID: 31233991

[69] López-Martínez C Serrano-Ortega N Moreno-Cámara S Del-Pino-Casado R. Association between sense of coherence associated with mental health in caregivers of older adults. Int J Environ Res Public Health. (2019) 16:3800. doi: 10.3390/ijerph16203800, PMID: 31601018 PMC6843852

[70] Ragger K Hiebler-Ragger M Herzog G Kapfhammer HP Unterrainer HF. Sense of coherence is linked to post-traumatic growth after critical incidents in Austrian ambulance personnel. BMC Psychiatry. (2019) 19:89. doi: 10.1186/s12888-019-2065-z, PMID: 30866860 PMC6417083

[71] Hogh A Mikkelsen EG. Is sense of coherence a mediator or moderator of relationships between violence at work and stress reactions? Scand J Psychol. (2005) 46:429–37. doi: 10.1111/j.1467-9450.2005.00474.x, PMID: 16179025

[72] Olsson G Hemström O Fritzell J. Identifying factors associated with good health and ill health: not just opposite sides of the same coin. Int J Behav Med. (2009) 16:323–30. doi: 10.1007/s12529-009-9033-9, PMID: 19288207

[73] Holmberg S Thelin A Lena-Stiernström L. Relationship of sense of coherence to other psychosocial indices. Eur J Psychol Assess. (2006) 20:227–36. doi: 10.1027/1015-5759.20.4.227

